# Leukotriene B₄ Metabolism and p70S6 Kinase 1 Inhibitors: PF-4708671 but Not LY2584702 Inhibits CYP4F3A and the ω-Oxidation of Leukotriene B₄ *In Vitro* and *In Cellulo*

**DOI:** 10.1371/journal.pone.0169804

**Published:** 2017-01-09

**Authors:** Anne-Sophie Archambault, Caroline Turcotte, Cyril Martin, Julie S. Lefebvre, Véronique Provost, Michel Laviolette, Nicolas Flamand

**Affiliations:** 1 Centre de recherche de l’Institut universitaire de cardiologie et de pneumologie de Québec, Québec City, Québec, Canada; 2 Département de médecine, Faculté de médecine, Université Laval, Québec City, Québec, Canada; Fundacao Oswaldo Cruz, BRAZIL

## Abstract

LTB_4_ is an inflammatory lipid mediator mainly biosynthesized by leukocytes. Since its implication in inflammatory diseases is well recognized, many tools to regulate its biosynthesis have been developed and showed promising results *in vitro* and *in vivo*, but mixed results in clinical trials. Recently, the mTOR pathway component p70S6 kinase 1 (p70S6K1) has been linked to LTC_4_ synthase and the biosynthesis of cysteinyl-leukotrienes. In this respect, we investigated if p70S6K1 could also play a role in LTB_4_ biosynthesis. We thus evaluated the impact of the p70S6K1 inhibitors PF-4708671 and LY2584702 on LTB_4_ biosynthesis in human neutrophils. At a concentration of 10 μM, both compounds inhibited S6 phosphorylation, although neither one inhibited the thapsigargin-induced LTB_4_ biosynthesis, as assessed by the sum of LTB_4_, 20-OH-LTB_4_, and 20-COOH-LTB_4_. However, PF-4708671, but not LY2584702, inhibited the ω-oxidation of LTB_4_ into 20-OH-LTB_4_ by intact neutrophils and by recombinant CYP4F3A, leading to increased LTB_4_ levels. This was true for both endogenously biosynthesized and exogenously added LTB_4_. In contrast to that of 17-octadecynoic acid, the inhibitory effect of PF-4708671 was easily removed by washing the neutrophils, indicating that PF-4708671 was a reversible CYP4F3A inhibitor. At optimal concentration, PF-4708671 increased the half-life of LTB_4_ in our neutrophil suspensions by 7.5 fold, compared to 5 fold for 17-octadecynoic acid. Finally, Michaelis-Menten and Lineweaver-Burk plots indicate that PF-4708671 is a mixed inhibitor of CYP4F3A. In conclusion, we show that PF-4708671 inhibits CYP4F3A and prevents the ω-oxidation of LTB_4_ *in cellulo*, which might result in increased LTB_4_ levels *in vivo*.

## Introduction

Leukotrienes (LT) are inflammatory lipid mediators derived from arachidonic acid. They participate in the inflammatory cascade in numerous conditions, notably asthma, rheumatoid arthritis, allergies and in host defense [1, 2]. They are mainly biosynthesized by leukocytes via the 5-lipoxygenase (5-LO) pathway. With the help of its activating protein, 5-LO metabolizes arachidonic acid into the unstable intermediate LTA_4_. LTA_4_ can subsequently be metabolized into LTB_4_ by the LTA_4_ hydrolase, or into LTC_4_ by the LTC_4_ synthase. Cysteinyl-LTs are well known for their role in asthma and bronchoconstriction, while LTB_4_ is more involved in leukocyte recruitment and activation. In humans, LTB_4_ can be further metabolized into 12-oxo-LTB_4_ by the LTB_4_ 12-hydroxydehydrogenase, or it can be ω-oxidized by the CYP4F3A [3–5]. The latter, which is mainly expressed in neutrophils, catalyzes the formation of 20-OH-LTB_4_, then 20-COOH-LTB_4_.

The recognized implication of LTB_4_ in inflammation makes it an attractive therapeutic target. However, the inhibition of LTB_4_ biosynthesis showed mixed results in clinical trials, despite promising results in mice models of inflammatory diseases [1, 2]. Among the numerous compounds tested, only the 5-LO inhibitor Zileuton has been approved as a treatment for asthma. Therefore, a better understanding of LTB_4_ metabolism and its regulation could lead to new therapeutic approaches.

Two recent studies linked the mTOR pathway component p70S6 kinase 1 (p70S6K1) to LTC_4_ biosynthesis, showing that p70S6K1 could phosphorylate the LTC_4_ synthase, hence modulating its activity [6, 7]. Herein, we sought to determine whether p70S6K1 could also modulate LTB_4_ biosynthesis and metabolism. We thus evaluated the impact of two selective p70S6K1 inhibitors, PF-4708671 [8] and LY2584702 [9], on the biosynthesis of LTB_4_ and its metabolites in human neutrophils.

## Materials and Methods

### Material

Lymphocyte separation medium, aprotinin, dimethyl sulfoxide (DMSO) and solvents for HPLC and LC/MS were purchased from Thermo Fisher Scientific (Ottawa, Ontario, Canada). Dextran, adenosine deaminase, leupeptin and potassium phosphate were obtained from Sigma-Aldrich Canada (Oakville, Ontario, Canada). HBSS was purchased from Wisent Bioproducts (St-Bruno, Quebec, Canada). 19-OH-prostaglandin (PG) B_2_, PGB_2_, PGB_2_-D_4_ and 17-octadecynoic acid (17-ODYA) were purchased from Cayman Chemicals (Ann Arbor, Michigan, USA). PF-4708671 was obtained from Abcam (Cambridge, Massachusetts, USA) and LY2584702 from Selleckchem (Houston, Texas, USA). Thapsigargin was obtained from Tocris Bioscience (Ellisville. Missouri, USA). LTB_4_ was a generous gift from Dr Louis Flamand (Université Laval, Québec City, Canada). Recombinant CYP4F3A and the NADPH regenerating system were purchased from Corning (Corning, New York, USA). Protease and phosphatase inhibitor cocktail tablets were purchased from Roche (Laval, Quebec, Canada). Primary (anti-phospho-S6 #2211 and anti-S6 #2317) and secondary antibodies were obtained from Cell Signaling (Danvers, Massachusetts, USA). The enhanced chemiluminescent (ECL) substrate was obtained from Millipore Canada Ltd (Toronto, Ontario, Canada).

### Preparation and utilization of adenosine deaminase

Adenosine deaminase was prepared and utilized exactly as described before [10].

### Isolation of human neutrophils and cell stimulations

Human neutrophils were isolated from the peripheral blood of healthy volunteers, without consideration for gender, as described before [11]. For the experiments investigating the impact of p70S6 kinase inhibitors on LTB_4_ biosynthesis and LTB_4_ half-life, pre-warmed human neutrophil suspensions (37°C, 5 million cells/ml in HBSS containing 1.6 mM CaCl_2_) were incubated with PF-4708671, LY2584702 or vehicle (DMSO) for 5 minutes, then stimulated with 100 nM thapsigargin for 10 minutes or 1 μM LTB_4_ for different times (see Figures). For experiments in which the reversibility of PF-4708671 and 17-ODYA were assessed, pre-warmed human neutrophil suspensions were incubated with PF-4708671, 17-ODYA or vehicle (DMSO) for 5, 15 or 30 minutes. Cells were centrifuged (350 × *g*) and the pellets were suspended in HBSS-CaCl_2_ or autologous plasma for 20 minutes. Cells were washed 3 times with warm HBSS-CaCl_2_ before adding 1 μM LTB_4_ for 20 minutes.

### Analysis of LTB_4_ and its ω-oxidation products

Incubations were stopped by adding 1 volume of a cold (-30°C) stop solution (MeOH/MeCN, 1/1, v/v) containing 12.5 ng of both 19-OH-PGB_2_ and PGB_2_ as internal standards. The samples were placed at -30°C overnight to allow protein denaturation and then centrifuged (1000 × g, 10 minutes, 4°C). The resulting supernatants were analyzed by reversed-phase HPLC using a Shimadzu HPLC System (Shimadzu corporation, Kyoto, Japan) and an on-line extraction procedure based on a method from Borgeat *et al* [12]. In brief, samples were diluted 1/3 with water, then injected and loaded onto a C8 precolumn (Aquapore Octyl 7 μM, PerkinElmer, Waltham, USA) using water containing 0.01% phosphoric acid during 4.25 minutes. The C8 precolumn was then switched on-line with the HPLC analytical column (Accucore^™^ C18, 50 × 4.6 mm, 2.6 μm, ThermoFisher Scientific, Ottawa, Canada) and elution was performed using a discontinuous binary gradient with solvent A and solvent B. Solvent A consisted of MeOH/MeCN/H_2_O at a 44/11/45 ratio (v/v/v) plus 0.01% AcOH and DMSO. Solvent B consisted of MeOH/MeCN/H_2_O at a 63/32/5 ratio (v/v/v) plus 0.01% AcOH. The gradient was: 0–15% B from 4.25 to 5.25 minutes; 15–70% B from 5.25 to 12 minutes; 70–100% B from 12 to 12.30 minutes and held at 100% during 4 minutes. Column then was re-equilibrated into 15% solvent B during 5 minutes before the next sample was injected. Using this method, the retention times were 5.3 minutes for 19-OH-PGB_2_, 6.4 minutes for 20-COOH-LTB_4_, 6.7 minutes for 20-OH-LTB_4_, 8.9 minutes for PGB_2_, 9.8 minutes for 6Z-LTB_4_, 9.9 minutes for 6Z-12epi-LTB_4_, 10.1 minutes for LTB_4_, and 12.5 minutes for 5-HETE. Internal standards and LTs were detected by UV at 270 nm while 5-HETE was detected at 235 nm. Leukotrienes represent the sum of LTB_4_, 20-OH-LTB_4_ and 20-COOH-LTB_4_.

### *In vitro* CYP4F3A assay

Human recombinant CYP4F3A (5 pg/ml) in potassium phosphate buffer (100 mM, pH 7.4) containing a NADPH generating system (glucose-6-phosphate, NADP+, glucose-6-phosphate dehydrogenase, MgCl_2_) was warmed at 37°C then incubated for 5 minutes with inhibitors or vehicle (DMSO). LTB_4_ (1-20 μM) then was added and reactions were stopped at different times with 5 volumes of a cold stop solution. LTB_4_ and its ω-oxidation products were quantified by HPLC as described in methods. The initial reaction rate for each LTB_4_ concentration was determined. The maximal velocity (v_max_) and the Michaelis-Menten constant (K_M_) were calculated for each concentration of PF-4708671 to assess the type of inhibition, using non-linear regression of the Michaelis-Menten graph with the Graphpad Prism 7 Software (GraphPad Software, Inc., La Jolla, California, USA). The Michaelis-Menten graph was also linearized using the Lineweaver-Burk (double reciprocal) plot.

### Immunoblot

Pre-warmed neutrophil suspensions (37°C, 5 million cells/ml in HBSS containing 1.6 mM CaCl_2_) were stimulated with 100 nM of thapsigargin or N-Formylmethionine-leucyl-phenylalanine (fMLP) for 5 minutes. PF-4708671, LY2584702 or vehicle were added 5 minutes before stimulation. Incubations were stopped using 1 volume of cold (4°C) incubation buffer. The suspensions were centrifuged (350 x g, 5 min, 4°C) and then lysed in a cold (4°C) hypotonic buffer (10 mM Tris-HCl, 10 mM NaCl, 3 mM MgCl2, 1 mM EDTA, pH 7.4) containing 0.1% NP-40, protease inhibitors (10 μg/ml aprotinin, 10 μg/ml leupetin, 1 mM PMSF, protease inhibitor cocktail tablets), 2 mM diisopropyl fluorophosphate (DFP) and phosSTOP. Cells were vortexed for 15 seconds, then immediately solubilized in electrophoresis sample buffer (62.5 mM Tris-HCl, pH 6.8, 10% glycerol, 0.01% bromophenol blue, 5% β-mercaptoethanol, 2% SDS) and boiled for 10 minutes. Proteins were loaded on a 12% polyacrylamide gel for electrophoresis, and transferred onto a PVDF membrane. Membranes were blocked using TBS/Tween buffer containing 5% w/v skim milk and incubated overnight at 4°C with primary antibodies (anti-phospho-S6 #2211 and anti-S6 #2317, Cell Signaling) in TBS/Tween containing 5% skim milk. HRP-linked secondary antibodies and ECL substrate were used for detection.

### Quantification of PF-4708671 by LC-MS/MS

Incubations were stopped by adding one volume of cold (-30°C) MeOH + 0.01% acetic acid containing 2 ng of PGB_2_-D_4_ as an internal standard. The samples were placed at -30°C overnight to allow protein denaturation and then centrifuged (1000 × g, 10 minutes). The resulting supernatants were collected and diluted with water + 0.01% acetic acid to obtain a final MeOH concentration ≤ 10%. Lipids were extracted from the samples using solid phase extraction cartridges (Strata-X Polymeric Reversed Phase, 60 mg/1ml, Phenomenex). The eluate was dried under a stream of nitrogen and reconstituted in 50 μl of MeOH. 1 μl was injected onto an HPLC column (Kinetex C8, 150 × 2.1 mm, 2.6 μm, Phenomenex) and eluted at a flow rate of 500 μl/min with a linear gradient using 0.1% formic acid (solvent A) and acetonitrile containing 0.1% formic acid (solvent B). The gradient lasted 20 minutes, starting at 10:90 (A:B) a finishing at 90:10 (A:B). The HPLC system was interfaced with the electrospray source of a Shimadzu 8050 triple quadrupole mass spectrometer and mass spectrometric analysis was done in the negative ion mode using multiple reaction monitoring for the specific mass transition *m/z* 389.10 → 197.95.

### Statistical analyses

Data are represented as the mean ± S.D. All calculations were done using the Graphpad Prism 7 Software.

### Ethics

This study was approved by the local ethics committee (Comité d’éthique de la recherche de l’Institut universitaire de cardiologie et de pneumologie de Québec) and all subjects signed a consent form.

## Results

### p70S6 kinase 1 inhibitors do not inhibit the biosynthesis of LTB_4_ but PF-4708671 prevents further LTB_4_ metabolism

**Fig 1:**
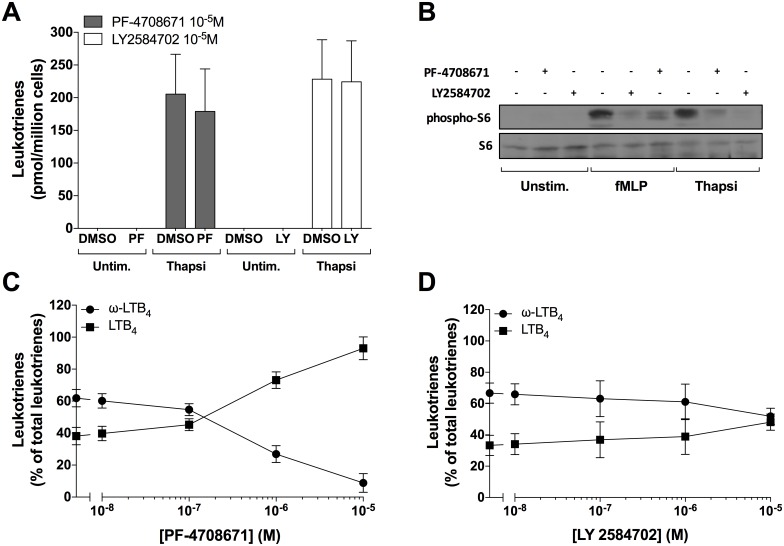
Impact of the p70S6K1 inhibitors on LTB_4_ biosynthesis and ω-oxidation in neutrophil suspensions. **A-D)** Pre-warmed human neutrophil suspensions (37°C, 5 million cells/ml in HBSS containing 1.6 mM CaCl_2_) were incubated with PF-4708671, LY2584702 or vehicle (DMSO) for 5 minutes, then stimulated with 100 nM thapsigargin for 10 minutes. **A,C,D**) Samples were processed and analyzed for LTB_4_ biosynthesis as described in methods. Data are the mean (± S.D) of 5 independent experiments, each performed in duplicate. **A**) Leukotrienes represent the sum of LTB_4_, 20-OH-LTB_4_ and 20-COOH-LTB_4_. **C,D**) ω-LTB_4_ represents the sum of 20-OH-LTB_4_ and 20-COOH-LTB_4_. **B**) Samples were processed and analyzed for S6 and phospho-S6 content as described in methods. Data are from one experiment, representative of three.

In the first series of experiments, we investigated whether p70S6K1 inhibitors modulated LTB_4_ biosynthesis. As shown in Fig 1A, PF-4708671 and LY2584702 at a concentration of 10 μM did not stimulate nor inhibit the thapsigargin-induced LTB_4_ biosynthesis. At this concentration, both compounds inhibited the phosphorylation of S6 induced by thapsigargin or fMLP (Fig 1B), indicating that they were both efficiently inhibiting the p70S6K1. However, we noticed that 10 μM PF-4708671 prevented the metabolism of LTB_4_ into 20-OH-LTB_4_ and 20-COOH-LTB_4_ in thapsigargin-stimulated neutrophils with an IC_50_ of ~800 nM (Fig 1C). This led to a decrease in 20-OH- and 20-COOH-LTB_4_ levels, and an increase in LTB_4_ levels. In contrast, LY2584702 did not prevent the metabolism of LTB_4_ into 20-OH- and 20-COOH-LTB_4_ in thapsigargin-stimulated neutrophils (Fig 1D).

**Fig 2:**
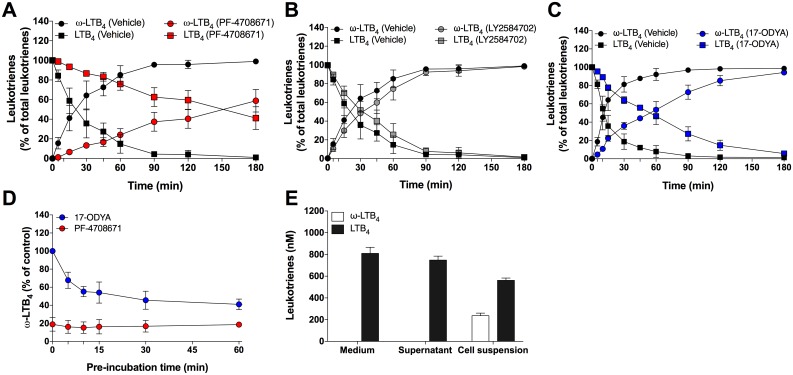
Impact of p70S6K1 and CYP4F3A on the half-life of LTB_4_ *in cellulo*. Pre-warmed human neutrophil suspensions (37°C, 5 million cells/ml in HBSS containing 1.6 mM CaCl_2_) were incubated 5 minutes with vehicle (DMSO), **A)** PF-4708671 (30 μM), **B)** LY2584702 (10 μM), or 30 minutes with **C)** 17-ODYA (30 μM), then treated with 1 μM of LTB_4_ for the indicated times. **D)** PF-4708671 (30 μM) or 17-ODYA (30 μM) were added to neutrophil suspensions for the indicated times before adding 1 μM LTB_4_ for 20 minutes. **E)** LTB_4_ (1 μM) was added for 20 minutes to either incubation medium, neutrophil supernatants (incubation medium incubated with neutrophils during 30 minutes) or neutrophil suspensions. **A-E)** Samples then were processed and analyzed for LTB_4_ biosynthesis as described in methods. ω-LTB_4_ represents the sum of 20-OH-LTB_4_ and 20-COOH-LTB_4_. Data are the mean (± S.D) of 4-5 independent experiments, each performed in duplicate.

We next determined if PF-4708671 could also inhibit the degradation of exogenously added LTB_4_ to 20-OH- and 20-COOH-LTB_4_ by performing kinetic experiments in which neutrophils were incubated with 1 μM LTB_4_. PF-4708671 increased the half-life of LTB_4_ by 7.5 fold, from ~20 minutes to ~150 minutes (Fig 2A). In contrast, LY2584702 did not significantly modulate LTB_4_ half-life (Fig 2B). In comparison, the CYP4F3A inhibitor 17-ODYA, previously shown to inhibit the metabolism of LTB_4_ into 20-OH-LTB_4_ in human neutrophils [13], increased LTB_4_ half-life by 5 fold, from ~10 minutes to ~50 minutes (Fig 2C). In these experiments, neutrophils were treated with PF-4708671 and 17-ODYA during 5 and 30 minutes respectively before the addition of LTB_4_. This is because PF-4708671 exerted its inhibitory constraint almost instantaneously while the optimal inhibitory effect of 17-ODYA was observed after 30 minutes (Fig 2D). Of note, the conversion of LTB_4_ into 20-OH- and 20-COOH-LTB_4_ only occured when neutrophils were present in the incubation media (Fig 2E). Indeed, LTB_4_ was not transformed into ω-LTB_4_ in our incubation medium (HBSS) or in a neutrophil supernatant, but was efficiently ω-oxidized in neutrophil suspensions, supporting the fact that the ω-oxidation of LTB_4_ is an intracellular event.

### PF-4708671 is a mixed inhibitor of CYP4F3A

**Fig 3:**
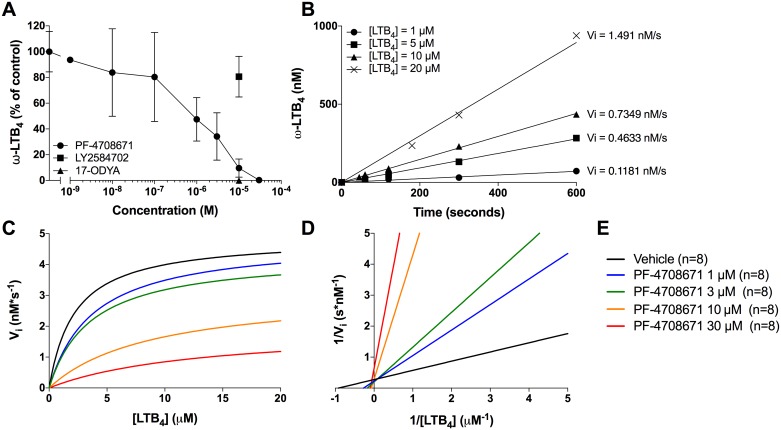
Impact of PF-4708671 on human recombinant CYP4F3A activity. **A)** Pre-warmed human recombinant CYP4F3A in phosphate buffer containing a NADPH regenerating system was incubated with increasing concentrations of PF-4708671 for 5 minutes, with LY2584702 (10^-5^M) for 5 minutes or with 17-ODYA (10^-5^M) for 30 minutes before the addition of 1 μM LTB_4_ for 1 minute. **B)** Representative time curves of the ω-oxidation of LTB_4_ par CYP4F3A. Initial rates were calculated by linear regession. **C)** Michaelis-Menten kinetics of the inhibition of CYP4F3A by PF-4708671. Human recombinant CYP4F3A was incubated with increasing concentrations of PF-4708671 before the addition of LTB_4_ at different concentrations. LTB_4_, and ω-LTB_4_ (20-OH-LTB_4_ and 20-COOH-LTB_4_) were quantified by HPLC as described in methods and represent the addition of 20-OH-LTB_4_ and 20-COOH-LTB_4_. Data are shown as the mean (± S.D) of 8 independent experiments. **D)** Data from the Michaelis-Menten graph were transformed using the Lineweaver-Burk plot (double reciprocal plot). **E)** Legend for C and D.

The distinct inhibitory profiles of PF-4708671 and LY2584702 on LTB_4_ metabolism raised the possibility that PF-4708671 was a CYP4F3A inhibitor. We thus tested this hypothesis by comparing the effect of p70S6K1 inhibitors with the CYP4F3A inhibitor 17-ODYA on human recombinant CYP4F3A activity. Human recombinant CYP4F3A was incubated with increasing concentrations of PF-4708671 or 17-ODYA for 5 and 30 minutes respectively, before the addition of 1 μM of LTB_4_ for 1 minute. PF-4708671 induced a concentration-dependent inhibition of ω-oxidation product formation (IC_50_ ~750 nM) while LY2584702 poorly affected this enzymatic conversion (Fig 3A). In other experiments, CYP4F3A was treated with increasing concentrations of PF-4708671 before the addition of various LTB_4_ concentrations. We then calculated the maximal rate of the reaction (v_max_) and the Michaelis-Menten constant (K_M_) during the steady-state of the reaction using the non-linear regression of the Michaelis-Menten graph. The representation of the data using either the Michaelis-Menten graph (Fig 3C) or the Lineweaver-Burk plot (Fig 3D) are consistent with a model of mixed inhibition. This mixed inhibition indicated that PF-4708671 was perhaps a substrate of CYP4F3A. However, LC-MS/MS analyses of PF-4708671 levels indicated that the compound remained stable in our neutrophil suspensions and in our enzymatic assay with recombinant CYP4F3A for up to 2 hours (data not shown).

### The inhibitory effect of PF-4708671 on LTB_4_ ω-oxidation is reversible

**Fig 4:**
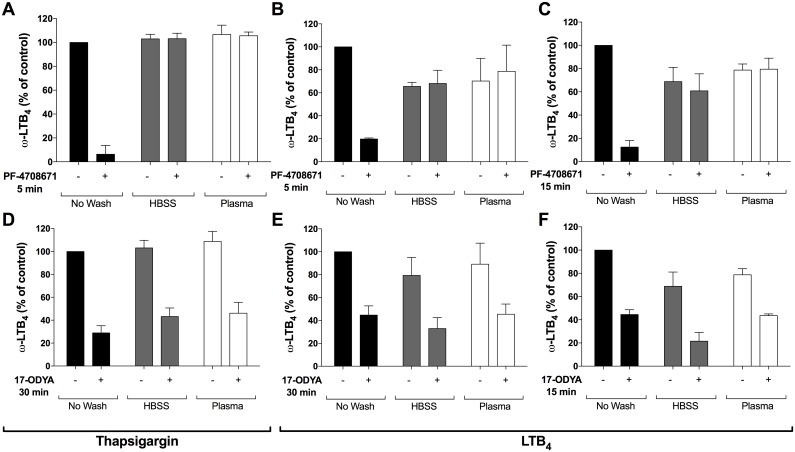
Removal of the inhibitory constraint exerted by CYP4F3A inhibitors on LTB_4_ ω-oxidation in neutrophils. Pre-warmed human neutrophil suspensions (37°C, 5 million cells/ml in HBSS containing 1.6 mM CaCl_2_) were incubated with **A,B)** 30 μM PF-4708671 or vehicle for 5 minutes, **C)** 30 μM PF-4708671 for 15 minutes, **D,E)** 30 μM 17-ODYA for 30 minutes, or **F)** 30 μM 17-ODYA for 15 minutes. Neutrophils were washed (or not) with autologous plasma or HBSS-CaCl_2_ as described in methods. **A,D)** 100 nM thapsigargin or **B,C,E,F)** 1 μM LTB_4_ were then added for 10 and 20 minutes, respectively. Samples then were processed and analyzed for 20-OH-LTB_4_ and 20-COOH-LTB_4_ as described in methods. Data are the mean (± S.D) of 4 independent experiments, each performed in duplicate.

Finally, we assessed if the inhibitory effect of PF-4708671 was reversible by comparing it with the irreversible CYP4F3A inhibitor 17-ODYA [13]. In these experiments, neutrophils were incubated with PF-4708671 for 5 minutes or 17-ODYA for 30 minutes (optimal incubation times) or with the inhibitors for 15 minutes (comparable incubation time). Then, neutrophils were either washed with HBSS-CaCl_2_, washed with autologous plasma or not washed at all, before treatement with either 100 nM thapsigargin or 1 μM LTB_4_. In absence of washing, both PF-4708671 and 17-ODYA inhibited the metabolism of LTB_4_ into 20-OH- and 20-COOH-LTB_4_ (Fig 4). Washing the PF-4708671-treated neutrophils with either HBSS-CaCl_2_ or autologous plasma restored the ability of neutrophils to metabolize endogenously formed and exogenously added LTB_4_ into 20-OH- and 20-COOH-LTB_4_ (Fig 4A–4C). In contrast, 17-ODYA-treated neutrophils that were washed with either HBSS-CaCl_2_ or autologous plasma remained incapable of metabolizing endogenously biosynthesized or exogenously added LTB_4_ into 20-OH- or 20-COOH-LTB_4_ (Fig 4D–4F). Furtermore, similar results were obtained whether we used the optimal incubation time for each inhibitor or the comparable incubation time (5 and 30 minutes for PF-4708671 and 17-ODYA *vs*. 15 minutes for each inhibitor). These experiments indicate that in contrast to the irreversible inhibitor 17-ODYA, the inhibitory constraint of PF-4708671 is easily removable and that the latter is a reversible inhibitor of the CYP4F3A enzyme.

## Discussion

Two recent studies have linked the mTOR pathway component p70S6K1 to LTC_4_ synthase function, providing a possible new way of regulating cysteinyl-LT biosynthesis [6, 7]. Thus, we wondered if p70S6K1 could play a similar role in the regulation of LTB_4_ synthesis. In that regard, our data show that **1)** the thapsigargin-induced LTB_4_ biosynthesis is not inhibited by p70S6K1 inhibitors; **2)** PF-4708671, but not LY2584702, inhibits the metabolism of LTB_4_ into 20-OH- and 20-COOH-LTB_4_ in a concentration-dependant manner; **3)** PF-4708671, but not LY2584702, inhibits human recombinant CYP4F3A; **4)** PF-4708671 is a reversible inhibitor of CYP4F3A; and **5)** PF-4708671 is a mixed inhibitor of CYP4F3A.

In this study, we aimed at documenting the inhibitory effect of p70S6K1 inhibitors on the ability of human neutrophils to metabolize LTB_4_ into 20-OH- and 20-COOH-LTB_4_. For that reason, we utilized an experimental model in which the priming of the arachidonic acid cascade and the 5-LO pathway were not involved, i.e. thapsigargin-stimulated neutrophils. In that experimental model, we could show that the ability of neutrophils to biosynthesize LTB_4_, which includes the sum of LTB_4_, 20-OH-LTB_4_, and 20-COOH-LTB_4_, was unchanged. This indicates that at the concentration utilized, the p70S6K1 inhibitors PF-4708671 and LY2584702 did not inhibit the enzymes involved in LTB_4_ biosynthesis in human neutrophils, notably cPLA_2_α, 5-LO, 5-LO-activating protein and LTA_4_ hydrolase [14–17]. However, it remains possible that the p70S6K1 inhibitors used in this study might impact LTB_4_ biosynthesis in human neutrophils when other signaling mechanisms are involved, notably those linked to the priming of LTB_4_ biosynthesis such as cytokines and TLR activation [18, 19], raising the possibility that PF-4708671, and possibly LY2584702, modulate LT biosynthetic pathways through multiple mechanisms of action.

We found that PF-4708671, but not LY2584702, inhibited the metabolism of LTB_4_ into 20-OH- and 20-COOH-LTB_4_ in a concentration-dependant manner. In that regard, PF-4708671 was more potent than 17-ODYA, a recognized CYP4F3A inhibitor [13]. Moreover, the effect of PF-4708671 was more pronounced that of 17-ODYA. While PF-4708671 increased the half-life of LTB_4_ in our human neutrophil suspensions by 7.5 fold, 17-ODYA increased it by 5 fold. This indicates that PF-4708671 might be a promising tool to develop specific and potent inhibitors of the CYP4F3A enzyme.

Given that the Michaelis-Menten and the Lineweaver-Burk plots indicated that PF-4708671 was a mixed inhibitor, we thought that perhaps PF-4708671 was a CYP4F3A substrate and was metabolized to some extent by the enzyme. However, this hypothesis was proven incorrect, as PF-4708671 was stable for at least 2 hours in the presence of either recombinant CYP4F3A or human neutrophils. The mixed inhibition we observed also raised the possibility that a contaminant in our commercial PF-4708671 preparation might also inhibit CYP4F3A. Although we cannot infirm that possibility, we tested three different batches of PF-4708671 which all yielded the same results, indicating that the effects we are documenting are very unlikely to be caused by a compound that is present in trace amounts.

This is not the first study to underscore a non-specific effect of PF-4708671. Another group reported an off-target effect of PF-4708671 in immortalized mouse fibroblasts, showing that PF-4708671 activates AMPK and inhibits the mitochondrial respiratory chain complex I, independently of p70S6K1 [20]. Moreover, PF-4708671 is used as a tool to study the mTOR pathway in various *in vivo* and *in vitro* studies, mainly in models of type 2 diabetes and cancer [21–33]. In light of our findings, it cannot be ruled out that some of the previously reported effects of PF-4708671 are caused by its lack of specificity and possibly increased LTB_4_ levels.

It was previously reported that LTB_4_ levels are increased in white adipose tissue, liver and muscles of mice fed with an high fat diet [34, 35]. Furthermore, mice lacking LTB_4_ receptor 1 are less susceptible to diet-induced insulin resistance [36, 37]. Therefore, using PF-4708671 in models of type 2 diabetes could increase LTB_4_ levels and possibly lead to an increased inflammation. However, in rodents, the functional orthologue of CYP4F3A is CYP4F18, which transforms LTB_4_ into 18-OH-LTB_4_ instead of 20-OH-LTB_4_ [38, 39]. While the activity of CYP4F18 towards LTB_4_ has been characterized, it is still unknown whether PF-4708671 exerts an inhibitory effect on CYP4F18 as well.

In conclusion, we demonstrate that PF-4708671 is a reversible CYP4F3A inhibitor preventing the metabolism of LTB_4_ into 20-OH- and 20-COOH-LTB_4_. In addition to characterizing a new compound that induces a sustained elevation in LTB_4_ levels, our data shed some light on the non-specific effects of a widely used p70S6K1 inhibitor. Given that it is more potent than the only currently available CYP4F3A inhibitor, PF-4708671 might be an helpful tool for the development of potent CYP4F3A inhibitors to study the regulation of LTB_4_ metabolism and its impact in inflammation.

## Supporting Information

S1 Appendix (XLSX)

## References

[1] Peters-Golden M, Henderson WR Jr. Leukotrienes. N Engl J Med. 2007;357(18):1841–54. 10.1056/NEJMra071371 17978293

[2] Flamand N, Mancuso P, Serezani CH, Brock TG. Leukotrienes: mediators that have been typecast as villains. Cell Mol Life Sci. 2007;64(19–20):2657–70. 10.1007/s00018-007-7228-2 17639273 PMC11136143

[3] Soberman RJ, Harper TW, Murphy RC, Austen KF. Identification and functional characterization of leukotriene B_4_ 20-hydroxylase of human polymorphonuclear leukocytes. Proc Natl Acad Sci U S A. 1985;82(8):2292–5. 2986111 10.1073/pnas.82.8.2292 PMC397543

[4] Kikuta Y, Kusunose E, Endo K, Yamamoto S, Sogawa K, Fujii-Kuriyama Y, et al A novel form of cytochrome P-450 family 4 in human polymorphonuclear leukocytes. cDNA cloning and expression of leukotriene B_4_ omega-hydroxylase. J Biol Chem. 1993;268(13):9376–80. 8486631

[5] Yokomizo T, Ogawa Y, Uozumi N, Kume K, Izumi T, Shimizu T. cDNA cloning, expression, and mutagenesis study of leukotriene B_4_ 12-hydroxydehydrogenase. J Biol Chem. 1996;271(5):2844–50. 8576264 10.1074/jbc.271.5.2844

[6] Esser J, Gehrmann U, Salvado MD, Wetterholm A, Haeggstrom JZ, Samuelsson B, et al Zymosan suppresses leukotriene C_4_ synthase activity in differentiating monocytes: antagonism by aspirin and protein kinase inhibitors. FASEB J. 2011;25(4):1417–27. 10.1096/fj.10-175828 21228223

[7] Ahmad S, Ytterberg AJ, Thulasingam M, Tholander F, Bergman T, Zubarev R, et al Phosphorylation of Leukotriene C_4_ Synthase at Serine 36 Impairs Catalytic Activity. J Biol Chem. 2016;291(35):18410–8. 10.1074/jbc.M116.735647 27365393 PMC5000086

[8] Pearce LR, Alton GR, Richter DT, Kath JC, Lingardo L, Chapman J, et al Characterization of PF-4708671, a novel and highly specific inhibitor of p70 ribosomal S6 kinase (S6K1). Biochem J. 2010;431(2):245–55. 10.1042/BJ20101024 20704563

[9] Tolcher A, Goldman J, Patnaik A, Papadopoulos KP, Westwood P, Kelly CS, et al A phase I trial of LY2584702 tosylate, a p70 S6 kinase inhibitor, in patients with advanced solid tumours. Eur J Cancer. 2014;50(5):867–75. 10.1016/j.ejca.2013.11.039 24440085

[10] Chouinard F, Turcotte C, Guan X, Larose MC, Poirier S, Bouchard L, et al 2-Arachidonoyl-glycerol- and arachidonic acid-stimulated neutrophils release antimicrobial effectors against E. coli, S. aureus, HSV-1, and RSV. J Leukoc Biol. 2013;93(2):267–76. 10.1189/jlb.0412200 23242611 PMC4995105

[11] Chouinard F, Lefebvre JS, Navarro P, Bouchard L, Ferland C, Lalancette-Hebert M, et al The endocannabinoid 2-arachidonoyl-glycerol activates human neutrophils: critical role of its hydrolysis and de novo leukotriene B_4_ biosynthesis. J Immunol. 2011;186(5):3188–96. 10.4049/jimmunol.1002853 21278347

[12] Borgeat P, Picard S, Vallerand P, Bourgoin S, Odeimat A, Sirois P, et al Automated on-line extraction and profiling of lipoxygenase products of arachidonic acid by high-performance liquid chromatography. Methods Enzymol. 1990;187:98–116. 2122189 10.1016/0076-6879(90)87014-t

[13] Shak S, Reich NO, Goldstein IM, Ortiz de Montellano PR. Leukotriene B_4_ omega-hydroxylase in human polymorphonuclear leukocytes. Suicidal inactivation by acetylenic fatty acids. J Biol Chem. 1985;260(24):13023–8. 2997155

[14] Flamand N, Lefebvre J, Surette ME, Picard S, Borgeat P. Arachidonic acid regulates the translocation of 5-lipoxygenase to the nuclear membranes in human neutrophils. J Biol Chem. 2006;281(1):129–36. 10.1074/jbc.M506513200 16275640

[15] Flamand N, Picard S, Lemieux L, Pouliot M, Bourgoin SG, Borgeat P. Effects of pyrrophenone, an inhibitor of group IVA phospholipase A_2_, on eicosanoid and PAF biosynthesis in human neutrophils. Br J Pharmacol. 2006;149(4):385–92. 10.1038/sj.bjp.0706879 16967052 PMC1978440

[16] Flamand N, Surette ME, Picard S, Bourgoin S, Borgeat P. Cyclic AMP-mediated inhibition of 5-lipoxygenase translocation and leukotriene biosynthesis in human neutrophils. Mol Pharmacol. 2002;62(2):250–6. 12130675 10.1124/mol.62.2.250

[17] Wetterholm A, Haeggstrom JZ, Samuelsson B, Yuan W, Munoz B, Wong CH. Potent and selective inhibitors of leukotriene A_4_ hydrolase: effects on purified enzyme and human polymorphonuclear leukocytes. J Pharmacol Exp Ther. 1995;275(1):31–7. 7562564

[18] Krump E, Boudreault S, Surette ME, Borgeat P. Effect of GM-CSF on leukotriene B_4_ synthesis in human neutrophils: facilitation of the priming effect by autologous plasma. Adv Exp Med Biol. 1997;400B:583–7. 9547607

[19] Surette ME, Dallaire N, Jean N, Picard S, Borgeat P. Mechanisms of the priming effect of lipopolysaccharides on the biosynthesis of leukotriene B_4_ in chemotactic peptide-stimulated human neutrophils. FASEB J. 1998;12(14):1521–31. 9806761 10.1096/fasebj.12.14.1521

[20] Vainer GW, Saada A, Kania-Almog J, Amartely A, Bar-Tana J, Hertz R. PF-4708671 activates AMPK independently of p70S6K1 inhibition. PLoS One. 2014;9(9):e107364 10.1371/journal.pone.0107364 25202971 PMC4159345

[21] Arnold N, Koppula PR, Gul R, Luck C, Pulakat L. Regulation of cardiac expression of the diabetic marker microRNA miR-29. PLoS One. 2014;9(7):e103284 10.1371/journal.pone.0103284 25062042 PMC4111545

[22] Rajan MR, Fagerholm S, Jonsson C, Kjolhede P, Turkina MV, Stralfors P. Phosphorylation of IRS1 at serine 307 in response to insulin in human adipocytes is not likely to be catalyzed by p70 ribosomal S6 kinase. PLoS One. 2013;8(4):e59725 10.1371/journal.pone.0059725 23565163 PMC3614923

[23] Shum M, Bellmann K, St-Pierre P, Marette A. Pharmacological inhibition of S6K1 increases glucose metabolism and Akt signalling in vitro and in diet-induced obese mice. Diabetologia. 2016;59(3):592–603. 10.1007/s00125-015-3839-6 26733005

[24] Choi HN, Jin HO, Kim JH, Hong SE, Kim HA, Kim EK, et al Inhibition of S6K1 enhances glucose deprivation-induced cell death via downregulation of anti-apoptotic proteins in MCF-7 breast cancer cells. Biochem Biophys Res Commun. 2013;432(1):123–8. 10.1016/j.bbrc.2013.01.074 23376066

[25] Grasso S, Tristante E, Saceda M, Carbonell P, Mayor-Lopez L, Carballo-Santana M, et al Resistance to Selumetinib (AZD6244) in colorectal cancer cell lines is mediated by p70S6K and RPS6 activation. Neoplasia. 2014;16(10):845–60. 10.1016/j.neo.2014.08.011 25379021 PMC4212257

[26] Hong SE, Kim EK, Jin HO, Kim HA, Lee JK, Koh JS, et al S6K1 inhibition enhances tamoxifen-induced cell death in MCF-7 cells through translational inhibition of Mcl-1 and survivin. Cell Biol Toxicol. 2013;29(4):273–82. 10.1007/s10565-013-9253-2 23942996

[27] Hong SE, Shin KS, Lee YH, Seo SK, Yun SM, Choe TB, et al Inhibition of S6K1 enhances dichloroacetate-induced cell death. J Cancer Res Clin Oncol. 2015;141(7):1171–9. 10.1007/s00432-014-1887-9 25471732 PMC11823794

[28] Khotskaya YB, Goverdhan A, Shen J, Ponz-Sarvise M, Chang SS, Hsu MC, et al S6K1 promotes invasiveness of breast cancer cells in a model of metastasis of triple-negative breast cancer. Am J Transl Res. 2014;6(4):361–76. 25075253 PMC4113498

[29] Qiu ZX, Sun RF, Mo XM, Li WM. The p70S6K Specific Inhibitor PF-4708671 Impedes Non-Small Cell Lung Cancer Growth. PLoS One. 2016;11(1):e0147185 10.1371/journal.pone.0147185 26771549 PMC4714881

[30] Song X, Dilly AK, Kim SY, Choudry HA, Lee YJ. Rapamycin-enhanced mitomycin C-induced apoptotic death is mediated through the S6K1-Bad-Bak pathway in peritoneal carcinomatosis. Cell Death Dis. 2014;5:e1281 10.1038/cddis.2014.242 24901052 PMC4607229

[31] Zhang Y, Wang Q, Chen L, Yang HS. Inhibition of p70S6K1 Activation by Pdcd4 Overcomes the Resistance to an IGF-1R/IR Inhibitor in Colon Carcinoma Cells. Mol Cancer Ther. 2015;14(3):799–809. 10.1158/1535-7163.MCT-14-0648 25573956 PMC4456303

[32] Segatto I, Berton S, Sonego M, Massarut S, D'Andrea S, Perin T, et al Inhibition of breast cancer local relapse by targeting p70S6 kinase activity. J Mol Cell Biol. 2013;5(6):428–31. 10.1093/jmcb/mjt027 23899505

[33] Ben-Hur V, Denichenko P, Siegfried Z, Maimon A, Krainer A, Davidson B, et al S6K1 alternative splicing modulates its oncogenic activity and regulates mTORC1. Cell Rep. 2013;3(1):103–15. 10.1016/j.celrep.2012.11.020 23273915 PMC5021319

[34] Horrillo R, Gonzalez-Periz A, Martinez-Clemente M, Lopez-Parra M, Ferre N, Titos E, et al 5-lipoxygenase activating protein signals adipose tissue inflammation and lipid dysfunction in experimental obesity. J Immunol. 2010;184(7):3978–87. 10.4049/jimmunol.0901355 20207999

[35] Mothe-Satney I, Filloux C, Amghar H, Pons C, Bourlier V, Galitzky J, et al Adipocytes secrete leukotrienes: contribution to obesity-associated inflammation and insulin resistance in mice. Diabetes. 2012;61(9):2311–9. 10.2337/db11-1455 22688342 PMC3425405

[36] Li P, Oh da Y, Bandyopadhyay G, Lagakos WS, Talukdar S, Osborn O, et al LTB_4_ promotes insulin resistance in obese mice by acting on macrophages, hepatocytes and myocytes. Nat Med. 2015;21(3):239–47. 10.1038/nm.3800 25706874 PMC4429798

[37] Spite M, Hellmann J, Tang Y, Mathis SP, Kosuri M, Bhatnagar A, et al Deficiency of the leukotriene B_4_ receptor, BLT-1, protects against systemic insulin resistance in diet-induced obesity. J Immunol. 2011;187(4):1942–9. 10.4049/jimmunol.1100196 21742977 PMC3150353

[38] Powell WS, Gravelle F. Metabolism of arachidonic acid by peripheral and elicited rat polymorphonuclear leukocytes. Formation of 18- and 19-oxygenated dihydro metabolites of leukotriene B_4_. J Biol Chem. 1990;265(16):9131–9. 2160957

[39] Christmas P, Tolentino K, Primo V, Berry KZ, Murphy RC, Chen M, et al Cytochrome P-450 4F18 is the leukotriene B_4_ omega-1/omega-2 hydroxylase in mouse polymorphonuclear leukocytes: identification as the functional orthologue of human polymorphonuclear leukocyte CYP4F3A in the down-regulation of responses to LTB_4_. J Biol Chem. 2006;281(11):7189–96. 10.1074/jbc.M513101200 16380383

